# Smoking is associated with increased eryptosis, suicidal erythrocyte death, in a large population-based cohort

**DOI:** 10.1038/s41598-024-53258-y

**Published:** 2024-02-06

**Authors:** Marvin Schmitt, Franz Ewendt, Alexander Kluttig, Rafael Mikolajczyk, F. Bernhard Kraus, Wim Wätjen, Paul-Christian Bürkner, Gabriele I. Stangl, Michael Föller

**Affiliations:** 1 Cluster of Excellence SimTech, University of Stuttgart, Stuttgart, Germany; 2 Institute of Agricultural and Nutritional Sciences, Martin Luther University Halle-Wittenberg, Halle (Saale), Germany; 3 Institute of Medical Epidemiology, Biometrics, and Informatics, Medical Faculty of the Martin Luther University Halle-Wittenberg, Halle (Saale), Germany; 4 Central Laboratory, Department for Laboratory Medicine, University Hospital Halle, Martin Luther University Halle-Wittenberg, 06120 Halle (Saale), Germany; 5 Department of Statistics, TU Dortmund University, Dortmund, Germany; 6 Department of Physiology, University of Hohenheim, Garbenstraße 30, 70599 Stuttgart, Germany

**Keywords:** Cardiovascular biology, Biomarkers

## Abstract

Smoking has multiple detrimental effects on health, and is a major preventable cause of premature death and chronic disease. Despite the well-described effect of inhaled substances from tobacco smoke on cell toxicity, the association between smoking and suicidal erythrocyte death, termed eryptosis, is virtually unknown. Therefore, the blood samples of 2023 participants of the German National Cohort Study (NAKO) were analyzed using flow cytometry analysis to determine eryptosis from fluorescent annexin V-FITC-binding to phosphatidylserine-exposing erythrocytes. Blood analyses were complemented by the measurement of hematologic parameters including red blood cell count, hematocrit, hemoglobin, mean corpuscular cell volume (MCV) and mean corpuscular hemoglobin (MCH). Eryptosis was higher in smokers than in non- and ex-smokers, and positively associated with the number of cigarettes smoked daily (*r* = 0.08, 95% CI [0.03, 0.12]). Interestingly, despite increased eryptosis, smokers had higher red blood cell indices than non-smokers. To conclude, smokers were characterized by higher eryptosis than non-smokers, without showing any obvious detrimental effect on classic hematological parameters.

## Introduction

Smoking is a well-described risk factor for disease and a major preventable cause of premature death and chronic diseases worldwide^1^. In particular, it negatively impacts the vasculature and induces impairment of microcirculation^2^. Inhaled tobacco smoke comprises substances such as carbon monoxide (CO), formaldehyde, acetaldehyde, benzopyrenes, and nicotine which can enter the circulation^3,4^. Most of these substances are toxic and can cause tissue damage by inducing oxidative stress and inflammation^5–7^. In addition, CO binds to hemoglobin to form carboxyhemoglobin which reduces oxygen-carrying capacity and oxygen utilization^8^. Although smoking has cytotoxic and hypoxic effects, there is comparatively limited data on the relationship between smoking and the red blood cell system. Interestingly, a few observational studies found tobacco smoking associated with higher red blood cell indices such as red blood cell count, hematocrit, hemoglobin, and mean corpuscular volume (MCV)^9,10^. On the other hand, in vitro data indicate that smoking causes erythrocyte death. It was shown that treatment of red blood cells with cigarette smoke extract or CO can induce eryptosis^11,12^, a certain form of programmed cell death that is similar to classical apoptosis in numerous aspects^13^. Increased eryptosis was also observed in a small study comparing 21 healthy male smokers with 21 non-smokers^14^. Hallmarks of eryptosis are phosphatidylserine externalization^13^, which can be assessed by measuring the binding of fluorescent annexin V-FITC in flow cytometry analysis, and cell shrinkage^15^. Excessive eryptosis can cause anemia but also the derangement of microcirculation by adhesion of phosphatidylserine-exposing erythrocytes to endothelial cells^16,17^. Based on the findings of several observational studies indicating an association between smoking and increased red blood cell indices with other data, primarily obtained from in vitro experiments, observing eryptosis and shrinkage of erythrocytes, the current study aimed to investigate whether smoking and smoking habits are linked to significant changes in eryptosis in a large population-based cohort. Additionally, in the case of increased eryptosis, the study aimed to assess whether eryptosis is associated with anemia-like conditions or if it is linked to elevated red blood cell indices, which are observed in other cohorts.

## Methods

### Population and study design

Blood samples from 2023 participants of the German National Cohort Study (NAKO) in the study center of Halle (Saale) were analyzed. The study design of the NAKO was described in detail elsewhere^18–21^, and the characteristics of the participants included in the current study were published previously ^22^. In brief, NAKO includes more than 205,000 adult participants across 18 study centers in different German states. The data collection and processing procedures are standardized and underlie extensive quality control protocols^20^.

### Ethics statement

The current study was approved by the ethical review committee of the study center at MLU Halle-Wittenberg (L3 Project: Processing number: 2013–22) and was conducted in accordance with the Declaration of Helsinki.

### Recruitment and data collection

Recruitment and data collection processes were described in detail elsewhere^22^. In brief, participants of this study were recruited during the regular first follow-up of NAKO participants from 04/10/2019 to 11/04/2021. During the informed consent process for NAKO, participants were asked if they were willing to participate in our subproject, and an additional 3 ml serum tube was collected. Out of a total of 3190 NAKO study participants examined during the study period an additional blood sample was obtained from 2174 individuals. Blood samples were collected from Monday to Thursday in the Halle study center and transferred to the Institute of Agricultural and Nutritional Sciences, where the blood samples were analyzed for eryptosis. Hematological parameters (erythrocyte count, hematocrit, hemoglobin, MCV, mean corpuscular hemoglobin concentration (MCH), and mean corpuscular hemoglobin concentration (MCHC)) were measured by the Central Laboratory of the University Hospital Halle (Saale).

### Analytical methods

#### Flow cytometry analysis of annexin V-FITC-binding

Eryptosis was analyzed in fresh blood samples (maximum 24 h old) of the study participants by assessing phosphatidylserine exposure of erythrocytes as published elsewhere^22^. Phosphatidylserine exposure was measured by determining erythrocyte annexin V-FITC-binding serving as a proxy measure for eryptosis. In brief, 5 µl blood was added to 200 µl Ringer solution (pH 7.4; containing 125 NaCl mM, 5 mM KCl, 1 mM MgSO_4_, 32 mM HEPES, 5 mM glucose, and 1 mM CaCl_2_) to isolate the red blood cells. After centrifugation at 1800 g for 5 min, the supernatant was removed, and the washing step was repeated twice. Annexin V-FITC (BD Biosciences, Franklin Lakes, NJ, USA) was used at a 1:500 dilution to stain the erythrocytes resuspended in 250 µl of annexin V-FITC buffer (Ringer solution with 5 mM CaCl_2_) for 20 min at room temperature under protection from light. Ten µl of these samples were analyzed by flow cytometry (Cytoflex, Beckman Coulter, Brea, CA, USA) in duplicate. Annexin V-FITC-fluorescence intensity was determined at an excitation wavelength of 488 nm and an emission wavelength of 530 nm. Eryptosis (%) expresses the percentage of annexin V-FITC-binding cells of the gated erythrocyte population.

#### Measurement of hematological parameters

Erythrocyte count, hematocrit, hemoglobin, MCV, MCH, and MCHC were measured in the Central Laboratory of the University Hospital Halle (Saale) from whole blood samples on a Sysmex XN-9000 hematological analyzer. All analyses on the Sysmex XN-9000 analyzer were carried out according to the manufacturer’s instructions and manuals, with routine maintenance and internal as well as external quality control procedures.

### Statistical analysis

All data analyses were performed with the *R* programming language for statistical computing^23^. Statistical models were implemented in the *brms* package^24^ as an interface to the probabilistic programming language *Stan*^25^ for gold-standard Bayesian inference. All analyses relied on weakly informative priors since the exploratory nature of this study implies a lack of prior information. All analyses used four MCMC chains, each with 1000 warmup samples, followed by 2000 draws from the posterior distribution. Each model was checked with appropriate diagnostics^25^. Unless specified otherwise, all results were reported as Bayesian posterior means with 95% credible intervals (CI). Mean comparisons used models without equal variance assumption, i.e., modeling separate variances per group. Throughout the manuscript, *r* denotes the Pearson correlation coefficient and *d* denotes Cohen’s effect size measure for mean comparisons. The data pre-processing protocol encompassed the removal of invalid blood samples (as described in^22^) and extreme eryptosis values (median ± 3 inter-quartile range on the log scale), resulting in a total sample size of *n* = 2023.

### Consent to participate

Written informed consent was obtained from all participants.

## Results

### Characteristics of the study participants

For the sample of 2023 subjects (970 male, 1053 female) included in our analysis, basic characteristics and hematological parameters are depicted in Table 1. Among the participants, 1000 subjects identified themselves as non-smokers (defined as persons who have never smoked during their lifetime), 418 as smokers, and 605 as ex-smokers.

**Table 1:** Characteristics of the study participants.

	*n*	Mean	SD	Median	IQR	Min	Max
Age (years)	2023	56.43	12.53	57.26	17.97	25.35	77.22
BMI (kg/m^2^)	2008	26.98	5.09	26.20	6.10	16.50	61.80
Eryptosis^a^ (%)	2023	0.97	0.68	0.77	0.56	0.16	5.99
Erythrocyte count (Tpt/l)	1994	4.58	0.42	4.55	0.57	2.99	6.33
Hematocrit (l/l)	1993	0.41	0.03	0.41	5.00	0.23	0.57
Hemoglobin (mmol/l)	1994	8.58	0.77	8.50	0.11	3.90	11.60
MCV (fl)	1994	90.18	4.08	90.10	0.80	70.00	114.90
MCH (fmol)	1994	1.88	0.10	1.88	0.11	1.27	2.44
MCHC (mmol/l)	1994	20.82	0.60	20.80	0.80	17.20	23.20
Cigarettes per day (smoker)	408	12.02	7.80	10.00	12.25	0.07	40
Pack years (smoker)	412	19.55	15.94	16.60	22.79	0.02	92
Abstinence, years (ex-smoker)	605	17.72	12.56	16.00	20.00	0.00	49.00
Abstinence age, years (ex-smoker)	605	36.00	11.78	34.00	18.00	12.00	70.00

^a^% annexin V-FITC-binding erythrocytes.

*Tpt* Teraparticle, *MCV* Mean corpuscular volume, *MCH* Mean corpuscular hemoglobin, *MCHC* Mean corpuscular hemoglobin concentration, *IQR* Inter-quartile-range.

### Correlation between smoking behavior and eryptosis

**Figure 1:**
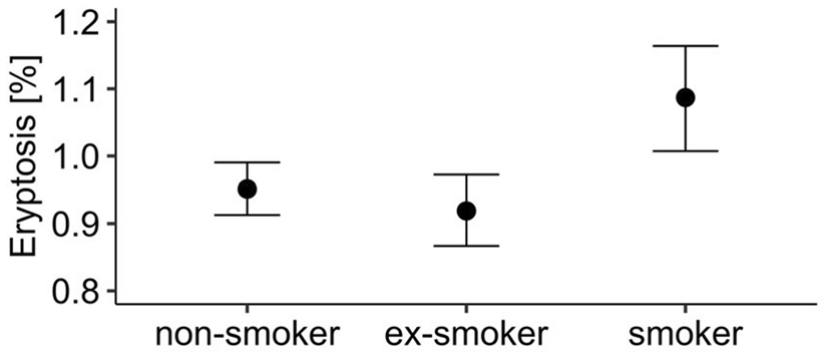
Eryptosis in non-smokers (*n* = 1000), ex-smokers (*n* = 605), and smokers (*n* = 418). Points indicate posterior means and bars indicate 95% CI. The figure was created using *R* programming language.

Group comparisons revealed that smokers showed moderately higher eryptosis as assessed from the percentage of phosphatidylserine-exposing erythrocytes than non-smokers and ex-smokers (Fig. 1), with posterior means [95% CI] as follows: non-smokers 0.95% [0.91%, 0.99%], ex-smokers 0.91% [0.86%, 0.96%], and smokers 1.08% [1.01%, 1.15%]. Specifically, smokers had a 14% higher percentage of eryptotic cells eryptosis than non-smokers (Cohen’s *d* = 0.19 [0.07, 0.31]), and a 19% higher percentage than ex-smokers (Cohen’s *d* = 0.22 [0.10, 0.35]). The percentage of eryptotic erythrocytes did not differ between ex-smokers and non-smokers (Cohen’s *d* = 0.05 [− 0.06, 0.14]).

**Figure 2:**
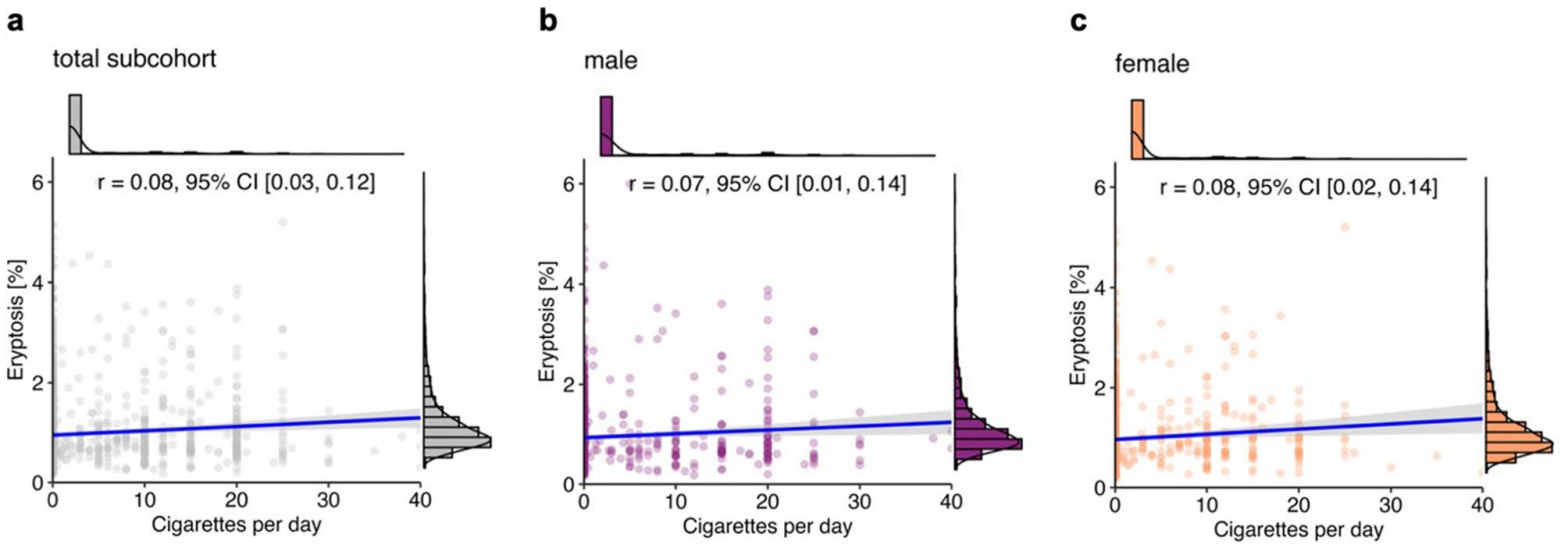
Correlation of eryptosis and the number of cigarettes per day in the total subcohort (*n* = 2013; **a**), and grouped by sex (**b**: 962 males; **c**: 1051 females). Lines indicate linear regression, and the blue ribbon around the regression line shows the uncertainty as 95% CI. The figure was created using *R* programming language.

Further analysis yielded a positive association between the number of cigarettes smoked daily and eryptosis rate (*r* = 0.08 [0.03, 0.12], Fig. 2a). Subgroup analysis of male (*r* = 0.07 [0.01, 0.14], Fig. 2b) and female subjects (*r* = 0.08 [0.02, 0.14], Fig. 2c) revealed a similar relationship between the numbers of cigarettes smoked daily and eryptosis in both sexes.

**Figure 3:**
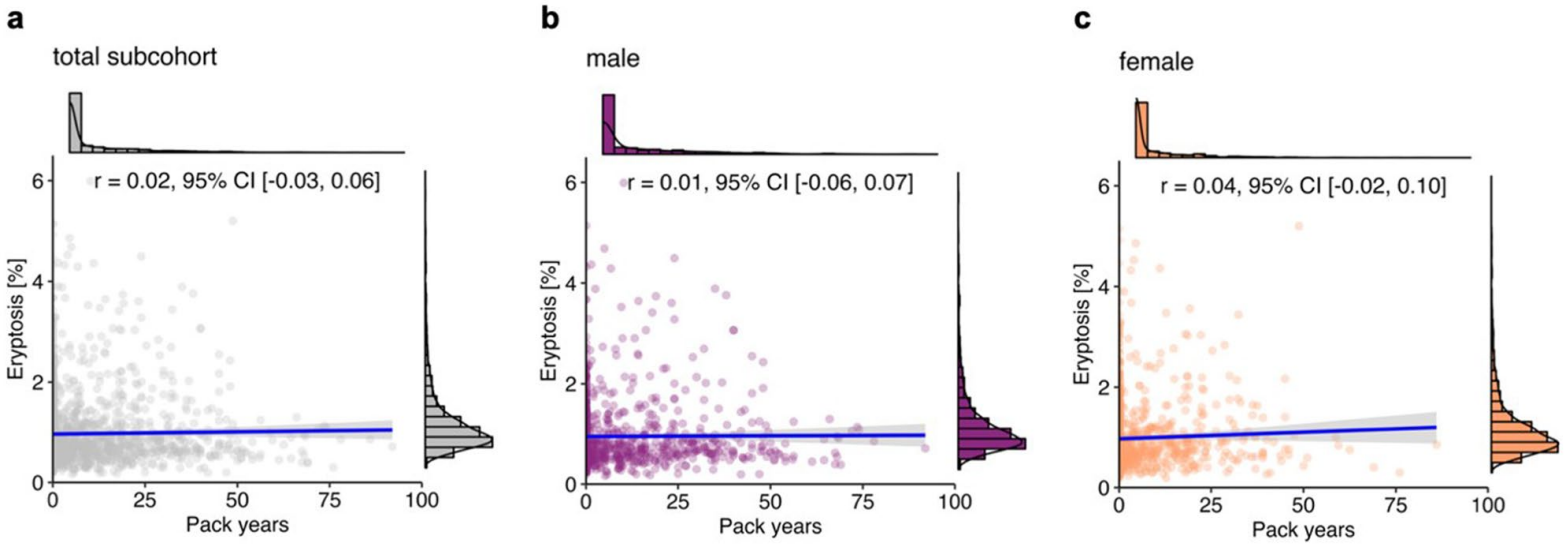
Correlation between eryptosis and pack years in the total cohort (*n* = 2016; **a**), and grouped by sex (**b**: 964 males; **c**: 1052 females). Lines indicate linear regression, and the gray ribbon around the regression line shows the uncertainty as 95% CI. The figure was created using *R* programming language.

As depicted in Fig. 3, the number of pack years, a cumulative exposure indicator of smoking burden, was not correlated with eryptosis in the total cohort (*r* = 0.02 [− 0.03, 0.06]; Fig. 3a) as well as in males (*r* = 0.01 [− 0.06, 0.07]; Fig. 3b) and females (*r* = 0.04 [− 0.02, 0.10]; Fig. 3c).

Next, we investigated whether the period of abstinence among ex-smokers showed an association with eryptosis. As a result, we found a negative association between the period of abstinence and eryptosis in ex-smokers (*r* =  − 0.13 [− 0.20, − 0.05]). Since longer smoking cessation was associated with lower eryptosis, we hypothesized that the age of smoking cessation might also influence eryptosis. Indeed, the age at which smoking had been stopped was an additional predictive factor for eryptosis (difference of expected log-predictive density ELPD = 2.9 with a standard error of SE = 2.4). This means that both factors, the age at which the subject stopped smoking and the time having passed since smoking cessation, are important prediction factors associated with eryptosis in ex-smokers.

### Hematological parameters

**Figure 4:**
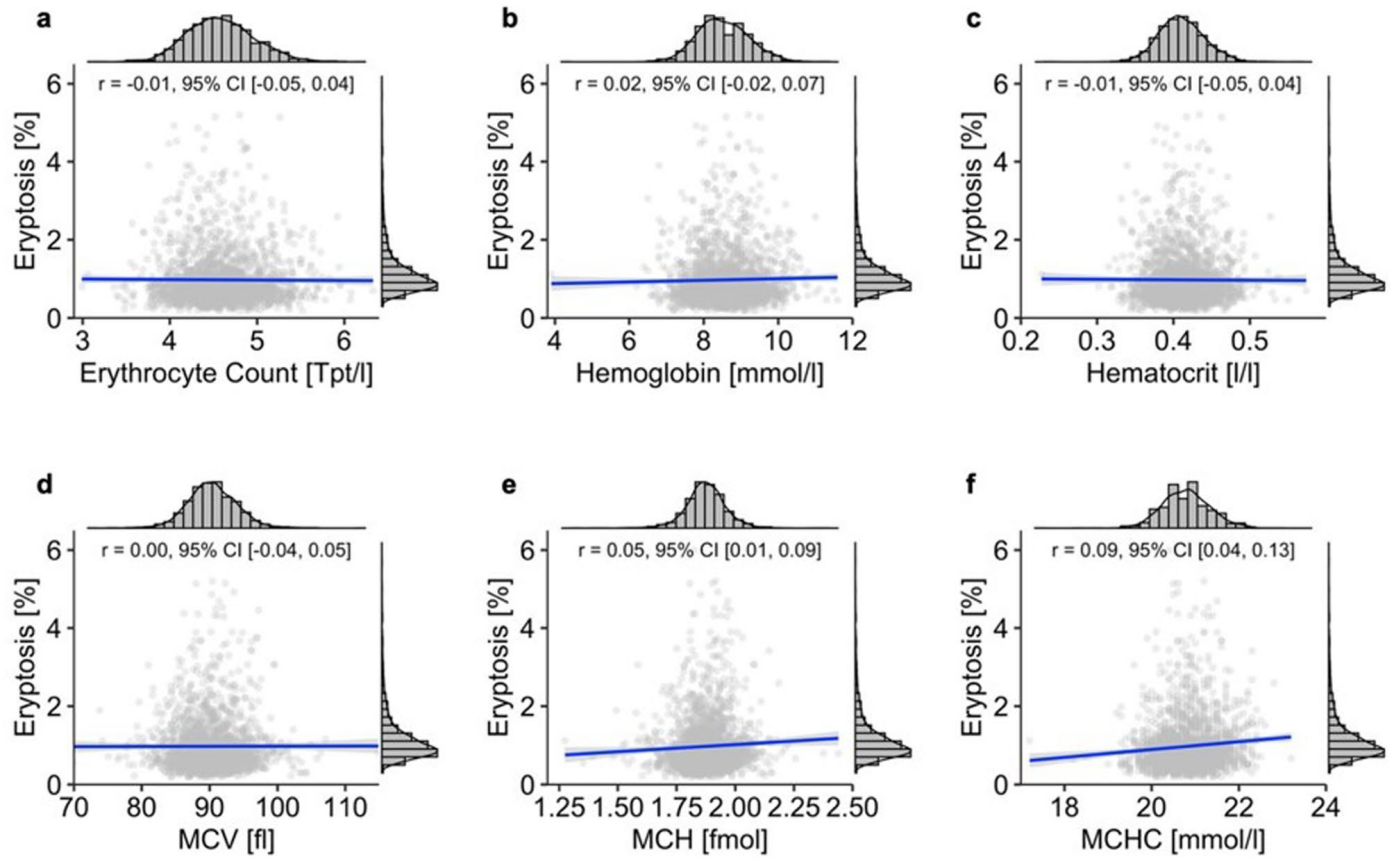
Correlation of eryptosis and hematological parameters of the total subcohort (cf. Table 1 for sample sizes of **a**–**f**). Lines indicate linear regression, and the blue ribbon around the regression line shows the uncertainty as 95% CI. The figure was created using *R* programming language. *Tpt* Teraparticle, *MCV* Mean corpuscular volume, *MCH* Mean corpuscular hemoglobin, *MCHC* Mean corpuscular hemoglobin concentration.

In addition, we investigated the relation of eryptosis and hematological parameters. As demonstrated in Fig. 4, eryptosis showed no association with erythrocyte count, hemoglobin, hematocrit, or MCV (Fig. 4a–d), while MCH and MCHC displayed a moderate positive correlation with eryptosis (Fig. 4e–f).

Table 2 shows the hematological parameters for current smokers, ex-smokers and non-smokers. Erythrocyte count and hematocrit did not differ between the three groups. In contrast, smokers exhibited higher values of hemoglobin, MCV, MCH, and MCHC than ex-smokers and non-smokers. As demonstrated in Table 3, there was no correlation between erythrocyte count and the number of cigarettes smoked per day, smoking duration and pack years within the group of current smokers. However, these parameters were moderately positively correlated with hematocrit, hemoglobin, MCV, and MCH, and negatively correlated with MCHC (Table 3).

**Table 2:** Hematological parameters by smoking status, i.e., smoker (*n* = 418), ex-smoker (*n* = 605), and non-smoker (*n* = 1000).

	Erythrocyte count (Tpt/l)	Hematocrit (l/l)	Hemoglobin (mmol/l)	MCV (fl)	MCH (fmol)	MCHC (mmol/l)
Smoker	4.58 [4.55, 4.62]	0.41 [0.41, 0.42]	8.71 [8.64, 8.78]	91.16 [90.73, 91.59]	1.90 [1.89, 1.91]	20.89 [20.83, 20.94]
Ex-smoker	4.58 [4.55, 4.61]	0.41 [0.41, 0.42]	8.60 [8.53, 8.66]	90.32 [90.00, 90.64]	1.88 [1.87, 1.89]	20.81 [20.77, 20.86]
Non-smoker	4.58 [4.55, 4.60]	0.41 [0.41, 0.41]	8.52 [8.47, 8.57]	89.69 [89.45, 89.93]	1.86 [1.86, 1.87]	20.80 [20.76, 20.83]

Point estimates indicate posterior means, values in brackets indicate 95% CI.

*Tpt* Teraparticle, *MCV* Mean corpuscular volume, *MCH* Mean corpuscular hemoglobin, *MCHC* Mean corpuscular hemoglobin concentration.

**Table 3:** Hematological parameters of smokers as a function of cigarettes smoked per day, smoking duration, and pack years.

	Erythrocyte count (Tpt/l)	Hematocrit (l/l)	Hemoglobin (mmol/l)	MCV (fl)	MCH (fmol)	MCHC (mmol/l)
Cigarettes per day	0.06 [− 0.04, 0.16]	0.17 [0.08, 0.26]	0.13 [0.03, 0.22]	0.16 [0.07, 0.25]	0.10 [0.00, 0.20]	− 0.10 [− 0.20, 0.01]
Smoking duration, years	0.02 [− 0.07, 0.11]	0.15 [0.05, 0.24]	0.08, [− 0.02, 0.17]	0.20 [0.10, 0.29]	0.10 [0.00, 0.19]	− 0.16 [− 0.25, − 0.07]
Pack years	0.05 [− 0.05, 0.14]	0.18 [0.09, 0.27]	0.12 [0.02, 0.21]	0.20 [0.11, 0.29]	0.12 [0.02, 0.21]	− 0.14 [− 0.24, − 0.05]

Point estimates indicate Pearson correlation coefficients, values in brackets indicate 95% CI.

*Tpt* Teraparticle, *MCV* Mean corpuscular volume, *MCH* Mean corpuscular hemoglobin, *MCHC* Mean corpuscular hemoglobin concentration.

## Discussion

Our study shows that in a large population-based cohort smokers exhibited moderately higher erythrocyte phosphatidylserine exposure than non-smokers and that the number of cigarettes smoked daily was positively correlated with this hallmark of eryptosis. Additionally, we demonstrated that the time of tobacco abstinence was associated with lower eryptosis with the age of smoking cessation having decisive impact.

The manifold detrimental effects of smoking are well characterized. The cardiovascular system is particularly affected^26^ as smoking induces vascular damage and may impair microcirculation^2,26^, conditions predisposing to myocardial infarction^27^ and stroke^28,29^. Since eryptotic red blood cells may adhere to vascular walls due to the interaction of phosphatidylserine on the erythrocyte surface with respective receptors on endothelial cells^30^, enhanced eryptosis may similarly compromize microcirculation^31^. The effect of smoking on eryptosis was, however, only moderate, and our study did not assess the contribution of eryptosis to an exacerbation of vascular pathologies. However, this contribution appears to be plausible.

Our study did not address the mechanism by which smoking triggers the cellular machinery ultimately resulting in eryptosis. Previous studies reported different mechanisms that may be effective: A pro-oxidative and pro-inflammatory milieu characterized by higher CRP levels and ROS formation, and reduced ROS scavenging capacity apparent from reduced erythrocyte glutathione levels may play a role^14^. Moreover, smokers are exposed to CO which has been demonstrated to directly stimulate eryptosis in vitro^11^. Finally, p38MAPK/Fas signaling has been shown to increase eryptosis in smokers^12^. Thus, it appears to be likely that these factors also account for higher eryptosis in stronger smokers observed in our study. However, other factors may also have an impact since the aforementioned ones may not fully clarify why quitting smoking earlier or for a longer period of time was linked to a reduction of eryptosis. It is tempting to speculate that general changes in lifestyle of individuals who quit smoking are in addition responsible for the observed effects.

An increased number of eryptotic red blood cells in smokers could in theory be expected to result in a reduced number of erythrocytes. However, our results are not indicative of anemia in smokers since erythrocyte count and other hematological parameters of smokers were not significantly different from those of non-smokers. However, we could not fully confirm the results from observational studies demonstrating higher red blood cell count, hematocrit, hemoglobin, and MCV in smokers than in non-smokers^9,10^. In line with those studies, we found moderately higher MCH and MCHC in smokers, in particular in those smoking a high number of cigarettes per day.

Higher eryptosis in smokers did not translate into a lower erythrocyte count. It must be kept in mind that enhanced erythropoiesis could, at least in theory, compensate higher eryptosis-dependent erythrocyte loss in smokers. Moreover, also a numerically small elevation in the percentage of phosphatidylserine-exposing erythrocytes can be expected to impede microcirculation due to the adhesion of these cells to the vascular wall. As a matter of fact, smokers do have impeded microcirculation^2^.

### Strengths and limitations

The strength of our investigation is the relatively large population-based sample with rigorous assessment of smoking. However, smoking was retrospectively reported and can be subject to reporting bias. Since prospective outcome data are not available yet, we are currently not able to study if higher eryptosis is indeed associated with an increased risk of cardiovascular diseases. Additionally, the underlying mechanism of enhanced eryptosis in heavier smokers could not be examined with our study design.

To conclude, our observational study revealed that smokers exhibit more eryptosis than non-smokers or ex-smokers. The higher proportion of eryptotic erythrocytes in smokers was not associated with a reduced number of erythrocytes and anemia, respectively.

## Data Availability

The datasets generated during and/or analyzed during the current study are not publicly available but are available from the corresponding author upon request.
